# Development of a Theory-Based mHealth App for Fatigue Management in Lupus: Human-Centered Design Approach

**DOI:** 10.2196/75399

**Published:** 2025-08-26

**Authors:** Anna Deck, Kiran Singh, Paula Caras, Amy LeClair, Monique Gore-Massy, Faye Chiu, Lucas Ogura Dantas, Timothy McAlindon, Sara Folta, Shanthini Kasturi

**Affiliations:** 1 School of Medicine, Tufts University, Boston, MA, United States; 2 Division of Rheumatology, Department of Medicine, Tufts Medical Center, 800 Washington Street, Boston, MA, 02111, United States, 1 617-636-5990; 3 Department of Medicine, Tufts Medical Center, Boston, MA, United States; 4 Patient Partner, West Orange, NJ, United States; 5 Patient Partner, New York, NY, United States; 6 Ambulomics, Sao Paolo, Brazil; 7 Division of Rheumatology, University of Massachusetts Chan Medical School, Worcester, MA, United States; 8 Friedman School of Nutrition, Tufts University, Boston, MA, United States

**Keywords:** systemic lupus erythematosus, fatigue, quality of life, mHealth, digital health, behavioral intervention, behavior change theory, human-centered design

## Abstract

**Background:**

Fatigue is a highly prevalent and debilitating symptom of systemic lupus erythematosus (SLE), significantly affecting the quality of life and employment of those living with the disease. Nonpharmacologic approaches, such as physical activity interventions, have shown promise in reducing fatigue but are often resource-intensive and lack grounding in behavior change theory. Mobile health (mHealth) technology offers a scalable approach to delivering behavioral interventions.

**Objective:**

This study describes the development of an mHealth app, grounded in behavior change theory, to support fatigue self-management in individuals with SLE by promoting physical activity.

**Methods:**

We used a human-centered design (HCD) approach to develop an mHealth app grounded in the self-determination theory of motivation and the social cognitive theory of behavior change. The process included two phases: (1) inspiration and (2) ideation. In the inspiration phase, key user needs were identified from focus groups of adults with SLE. During the ideation phase, a prototype was developed and iteratively refined based on feedback from additional individuals with SLE who participated in multiple rounds of semistructured interviews and online feedback surveys.

**Results:**

In the inspiration phase, 12 individuals participated in 2 focus groups and identified key priorities for the mHealth intervention, including symptom tracking (fatigue, pain, sleep, and physical activity), reliable educational content, social connection, and reminders. In the ideation phase, a prototype was developed based on these findings and refined through 2 rounds of user feedback interviews with 12 additional adults with SLE. Participants rated the features and format of the prototype favorably, with average scores ranging from 1.4 to 2.1 on a 5-point Likert scale (1=highest rating), and 78% (7/9) of interviewees reported they were likely or highly likely to use the app. Several themes around preferences for the app emerged from the interviews, including the importance of: (1) community and social connection, (2) accessibility and inclusion, (3) options for customization, (4) integration of the app with existing digital health tools, and (4) notifications for reminders and motivational messages. Based on this feedback, the prototype was refined, and a digital messaging feature was created. A library of 154 reminders and motivational messages was developed with input from 10 individuals with SLE who took part in a third round of interviews.

**Conclusions:**

Using an HCD approach, we developed an mHealth app tailored to the needs of individuals with SLE, integrating behavior change theory to support fatigue self-management. Through engagement with end users, we iteratively refined the app to address key priorities and enhance usability. This study demonstrates the feasibility of using HCD to develop an mHealth app grounded in behavior change theory and provides a model for creating rigorous digital health interventions for individuals with SLE and other chronic conditions.

## Introduction

Fatigue is the most common symptom of systemic lupus erythematosus (SLE) and affects 80%‐90% of individuals with this chronic autoimmune inflammatory disease [1]. Fatigue significantly impacts morbidity and is a leading cause of withdrawal from the workforce, with unemployment rates ranging from 40% to 60% among individuals with SLE [1-8]. People living with SLE consistently cite fatigue as their greatest unmet need; however, the relationship between SLE disease activity and fatigue is complex [9-15]. Studies suggest SLE-related fatigue is of multifactorial etiology, with the greatest contributors often being behavioral factors, namely poor sleep quality and physical inactivity; psychosocial factors, such as social isolation, and comorbid conditions, for example, depression and fibromyalgia [1,2,13-19].

Despite the significant burden of fatigue in individuals with SLE, there are limited effective treatment options available. Pharmacotherapy modifies fatigue mainly in those with highly active disease [20]. Nonpharmacologic approaches, such as physical activity interventions and education, have shown promise in reducing fatigue in individuals with SLE [21-29]. While a number of such interventions have been evaluated in SLE, these interventions often lack a grounding in and rigorous approach to behavior change theory [30-35]. In addition, such interventions are often resource-intensive or delivered in person, which can limit access and scalability.

Mobile health (mHealth) technology offers a platform to deliver behavioral interventions that lower barriers to participation and engagement. mHealth apps delivered through smartphones can provide tools for self-monitoring behavior, such as physical activity, by enabling real-time data capture and automated personalized feedback provided in individuals’ native context [36-38]. Interventions using mHealth-enabled behavior change strategies, such as self-monitoring and feedback via messaging, have been shown to facilitate physical activity, improve diet, and encourage other healthy behaviors across several medical conditions [39-43]. In addition, mHealth apps can overcome the logistical barriers of time and geography and offer resource and cost-effective alternatives to traditional in-person interventions.

Currently available mHealth apps for individuals with SLE are of limited quality and functionality. A 2020 systematic review of mHealth apps and technologies for SLE found that the majority of available apps scored poorly on the Mobile App Rating Scale, a validated tool for assessing the quality of apps based on engagement, functionality, aesthetics, information quality, and overall quality [44]. Existing apps were not developed with patient input and lacked evidence-based information and functionalities desired by patients, such as symptom and medication trackers and discussion groups and community features [44]. Of note, of the 20 apps identified in this review, many are no longer available or lack functional updates to be compatible with current technology. Individuals with SLE have a desire to use mHealth technology to aid with disease management, but there are few studies of mHealth interventions to improve health outcomes in SLE [45-47]. As such, there is a demonstrated need for mHealth tools for people with SLE that provide high-quality educational information, disease monitoring and management support, and social connection.

To address these gaps, we aimed to develop an mHealth-enabled behavioral intervention to reduce fatigue in individuals living with SLE by promoting physical activity. Rather than adapting an existing app, we sought to create a novel intervention from the ground up to address the unique behavioral and psychosocial contributors to fatigue in SLE—factors not targeted by mHealth tools designed for general populations. Recognizing the limitations of existing mHealth apps, our approach integrates behavior change theory and follows a human-centered design (HCD) process to ensure usability and alignment with patient needs. By leveraging mHealth technology, this intervention seeks to overcome traditional barriers to access, providing a scalable solution for fatigue management in SLE. Here, we outline the development process of this app.

## Methods

### Study Design

#### Theoretical Framework

**Figure 1.**
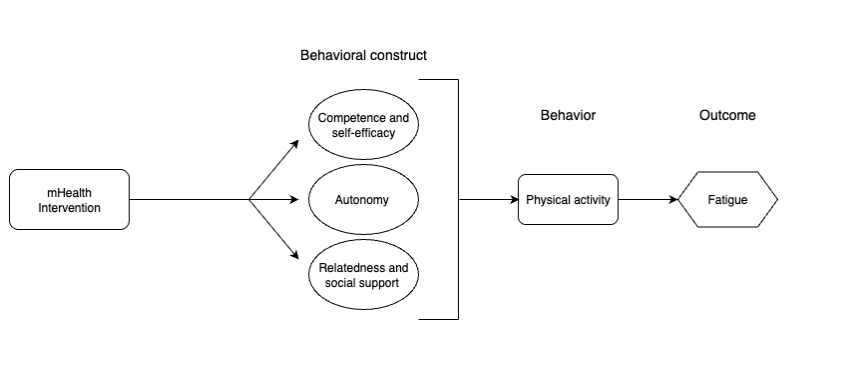
Theoretical framework for the intervention: self-determination and social cognitive theories. mHealth: mobile health.

The mHealth intervention is grounded in the self-determination theory (SDT) of motivation and social cognitive theory (SCT; Figure 1). SDT posits that successful behavior change hinges on addressing the key needs of relatedness, autonomy, and competence, while SCT emphasizes that behavior change relies on both environmental factors, such as social support, and cognitive factors, particularly self-efficacy [48-50]. Both SDT and SCT have been applied to a variety of health behaviors and provide a robust theoretical framework for promoting adherence to physical activity, the central behavior to be targeted in the mHealth intervention [51-53].

#### HCD Approach

A HCD approach was used to identify key stakeholder needs and develop the mHealth app for fatigue self-management. HCD places end users, in this case individuals living with SLE, at the center of the design process to create products that address key user needs and emphasize the whole user experience. This contrasts with traditional design methods, which typically develop products based primarily on expert opinion [54]. HCD consists of 3 stages: inspiration, ideation, and implementation. In the inspiration phase, key user needs are identified through immersive research, such as interviews and observation, and engagement with users. In the ideation phase, a prototype is developed and iteratively refined based on user feedback. During implementation, the final stage of HCD, the developed product is launched, integrated, and scaled in real-world settings [55,56]. This paper presents the findings from the first 2 stages, while the third phase will be reported separately following the completion of a pilot clinical trial of the intervention.

In addition to gathering feedback from end users through HCD methods, 2 patient partners (FC and MGM) were fully integrated into the research and development team. They actively contributed to every stage of development of the mHealth intervention, from conceptualization to implementation. These members of the research team participated in regular team meetings, ensuring that patient voices were heard throughout the process. They provided input on desired features, functionality, and format for the intervention, assisted with the development of feedback guides, and provided context to interpret the feedback provided by other end users. In cases of conflicting feedback, the patient partners shared their perspectives to support the decision-making process.

### HCD Phase 1: Inspiration

The inspiration phase of HCD focuses on understanding the needs, challenges, and preferences of the end user with the goal of developing a design solution that is human-centered. Our design challenge, to create an mHealth app to help people living with SLE manage their fatigue, emerged from formative and immersive work, including literature review, targeted conversations, and focus groups with patients with SLE.

#### Study Population

Participants were English-speaking adults (≥18 y of age) who had a diagnosis of SLE based on 2019 European League Against Rheumatism/American College of Rheumatology classification criteria and owned a smartphone [57]. Participants were recruited from the rheumatology clinic at Tufts Medical Center, an academic medical center in Boston, Massachusetts. Electronic health records were queried for patients with *ICD-9* (*International Classification of Diseases, Ninth Revision*) and/or *ICD-10* (*International Statistical Classification of Diseases, Tenth Revision*) diagnosis codes of SLE. Patients meeting inclusion criteria were contacted by telephone or in person at the time of a clinical visit and invited to participate in the study. Interested individuals were purposively sampled based on demographics (age, race, ethnicity, and insurance type) and disease duration to capture a diverse range of perspectives.

#### Data Collection

Two focus groups were conducted to elicit patient needs and preferences for the mHealth intervention for fatigue in SLE. A total of 12 individuals participated in these focus groups. Conversations were guided by a focus group guide (Multimedia Appendix 1), which probed desired content and features for the mHealth intervention, such as symptom and activity trackers, essential educational content, and practical strategies. Focus groups were led by an experienced moderator (AL) and were conducted in person. All focus groups were audio-recorded, transcribed, and deidentified.

#### Data Analysis

Participant demographics were analyzed using descriptive statistics. Focus group transcripts were independently reviewed by 2 investigators (AD and SK) using an integrated approach in which inductive codes emerging from line-by-line reading of the data were used alongside a priori codes generated from the theoretical frameworks of SDT and SCT. Codes were then reviewed together by both investigators and revised via a comparison and consensus approach, and themes were identified.

### HCD Phase 2: Ideation

The ideation phase of HCD aims to generate diverse ideas and potential solutions to the design challenge with the input of a multidisciplinary team of end users, designers/developers, and other stakeholders. Our development team consisted of individuals living with SLE, an app developer, a behavioral interventionalist, medical students, and a practicing rheumatologist. During this phase, app prototypes were generated and educational content for the intervention was developed. Here we discuss the development of the app prototype; development of the educational content is reported elsewhere [58].

#### Prototype Development

Potential features for the mHealth app identified during phase 1 were mapped to relevant behavioral constructs from SDT and SCT. A prototype of the app with desired features was developed using Figma (Figma Inc), a web-based collaborative design tool for creating and testing user interfaces and experiences for mobile apps. The prototype was reviewed and revised with input from the development team. The prototype was then iteratively refined through several rounds of feedback interviews with individuals with SLE.

#### Study Population

As in phase 1, participants in phase 2 were English-speaking adults diagnosed with SLE who owned smartphones. Participants were recruited once again through purposive sampling from the rheumatology clinic at Tufts Medical Center and additionally through the networks of our patient partners. There was no overlap of participants between phases 1 and 2.

#### Data Collection

A total of 12 individuals with SLE participated in 3 rounds of semistructured interviews to provide feedback on app prototypes. Conversations were led by an experienced facilitator (SK), using an interview guide (Multimedia Appendix 2) and modeled after think-aloud sessions [59,60]. Screenshots of the mHealth app from Figma were shared using Zoom (Zoom Video Communications) screen sharing capabilities. Each page of the app was displayed, and the functionality was explained. Interview participants were asked to share their impressions, including features they liked, aspects they would change, and additional ideas for improvement. The prototype was then modified based on feedback from the interviews, and a second round of interviews was conducted to evaluate the updated prototype. Interview participants also completed surveys rating the format and features of the prototypes (layout, color scheme, font, and graphics) and their likelihood to use the app using a numeric rating scale (1-5). A third round of interviews was conducted to evaluate app-based motivational and educational messaging that was developed in response to feedback from the first 2 cycles of interviews. Participants reviewed and rated the usefulness of the messages as part of the fatigue self-management intervention and provided open-ended feedback. Feedback was used to modify the messaging library and generate several new messages to address topics participants felt were missing. All surveys were administered using REDCap (Research Electronic Data Capture; Vanderbilt University). Interviews were conducted via Zoom web-based teleconference and were audio-recorded, transcribed, and deidentified.

#### Data Analysis

Participant demographics and survey responses were analyzed using descriptive statistics. Interview transcripts were independently reviewed by 2 investigators (AD and SK) who identified dominant themes guided by the theoretical frameworks of SDT and SCT. Themes were then reviewed and revised via a comparison and consensus approach. Transcripts of prototype feedback interviews were analyzed using a rapid analysis protocol and feedback capture grid [61,62].

### Ethical Considerations

The study protocol was reviewed and approved by the Tufts Health Sciences institutional review board (IRB number 13159 and number 00002106). Informed consent was obtained from all individuals participating in interviews or focus group discussions. Participants were compensated US $30 for each focus group or interview session.

## Results

### HCD Phase 1: Inspiration

Two focus groups including a total of 12 individuals with SLE (Table 1) were conducted.

Focus groups were about 30 minutes in duration and identified 4 key needs for the mHealth intervention (Table 2): social connection, educational content, tracking features, and reminders.

Participants expressed a desire for social connection and suggested ideas, including peer support features, chat functions, and connecting with health coaches. Participants emphasized the feeling of validation that came from participating in focus groups and felt that a peer support component in the app could offer similar validation. Participants also identified a need for reliable, evidence-based educational content within the app. Participants wanted general information about their SLE diagnosis and lupus-related fatigue, along with strategies for managing fatigue and improving sleep. They suggested content on physical activity, nutrition, and mental health and wellness. Finally, participants desired tracking features (such as pain, fatigue, activity, and sleep trackers) and reminders/notifications (such as prompts to be physically active) to help with SLE and fatigue self-management.

**Table 1.** Characteristics of focus group and semistructured interview participants (N=24).

Characteristic	Focus group participants (n=12)	Interview participants (n=12)	All participants (n=24)
Age (years), mean (SD)	44.3 (13.3)	42.4 (8.2)	43.4 (10.8)
SLE disease duration (years), mean (SD)	10.1 (8.8)	14.1 (7.2)	12.1 (8.1)
Sex, n (%)			
Female	12 (100)	10 (83)	22 (92)
Male	0 (0)	2 (17)	2 (8)
Race, n (%)			
White	3 (25)	4 (33)	7 (29)
Black or African-American	5 (42)	4 (33)	9 (38)
Asian	2 (17)	2 (17)	4 (17)
More than one race	2 (17)	2 (17)	4 (17)
Ethnicity, n (%)			
Hispanic	2 (17)	4 (33)	6 (25)
Non-Hispanic	10 (83)	8 (67)	18 (75)
Insurance type, n (%)			
Medicaid	6 (50)	1 (8)	7 (29)
Medicare	1 (8)	2 (17)	3 (13)
Third party/private	5 (42)	8 (67)	13 (54)
None	0 (0)	1 (8)	1 (4)
Education, n (%)			
High school graduate	2 (17)	2 (17)	4 (17)
Associate degree	3 (25)	1 (8)	4 (17)
Bachelor’s degree	4 (33)	6 (50)	10 (42)
Graduate degree	3 (25)	3 (25)	6 (25)
Employment, n (%)			
Part time	0 (0)	2 (17)	2 (8)
Full time	7 (58)	5 (42)	12 (50)
Self-Employed	1 (8)	2 (17)	3 (13)
On disability	2 (17)	3 (25)	5 (21)
Retired	1 (8)	0 (0)	1 (4)
Looking for work	1 (8)	0 (0)	1 (4)

**Table 2.** Inspiration phase: themes from focus groups.

User identified needs	Desired app features
Social connection	Peer support Chat with other app users Access to health coach
Educational content	Information about: Lupus Fatigue and Lupus Tips to manage fatigue Sleep Nutrition Mental health Reliable (with references) Accessible (appealing format and appropriate reading level)
Trackers	Pain Fatigue Sleep (quality and quantity) Physical activity
Reminders	Prompts to exercise

### HCD Phase 2: Ideation

#### Prototype Development

Drawing from the literature review and findings from qualitative work in phase 1, the following features were identified as essential components of the mHealth app: (1) physical activity goal-setting logs and trackers, (2) educational modules with self-assessment questions, (3) sleep trackers, (4) symptom trackers (fatigue, pain, and anxiety), (5) peer coach support, and (6) notifications. An initial prototype was developed with these functionalities. On review by the development team, the patient partners offered insight into visual accessibility (font size and color) and suggested several improvements. They recommended adding a daily “check-in” screen where users could rate how they were feeling, using weather pictograms, and their energy level, using spoons, a common analogy for describing energy in chronic disease [63]. They suggested adding free text journal features to this screen and the physical activity logs. Finally, they proposed reformatting the educational modules from text documents to infographics. These changes were incorporated into a second iteration of the prototype. Once the core components of the mHealth app were finalized, each feature was mapped to the relevant behavioral construct from SDT or SCT (Table 3).

**Table 3.** mHealth intervention behavioral construct mapping.

Behavioral construct from self-determination and social cognitive theories	mHealth^a^ intervention component
Competence/Self-Efficacy	
Knowledge	Educational modules (information on causes of fatigue, treatments for fatigue, benefits of physical activity in people living with lupus)
Behavioral skills/Self-regulation	Educational modules (information on how to be physically active, pacing/ “listen to your body”) Weekly physical activity goal setting Physical activity logs and progress visualization (self-monitoring) Tracker data (self-monitoring) Energy and movement journals Messages and notifications (feedback) Achievement page (feedback)
Outcome expectations	Educational modules (“what to expect when you start moving”)
Autonomy	Weekly physical activity goals (choice of type and amount of activity) Messages and notifications (autonomy supportive language) Educational modules (“find your why”; autonomy supportive language)
Social support/relatedness	Peer coach Group meetings via Zoom Participant chat group Program buddy
Barriers and opportunities	Messages and notifications (reminders)
Observational learning/normative beliefs	Educational modules (participant testimonials)

a mHealth: mobile health.

#### Prototype Iteration

A total of 12 individuals with SLE reviewed the prototype in 3 rounds of feedback interviews and surveys (Table 1). Participants gave high ratings to prototype features and format, and 78% (7/9) of participants reported being likely or highly likely to use the app (Table 4).

Interviews averaged 20‐25 minutes in length. The first round of interviews was analyzed using a rapid analysis protocol and feedback capture grids to categorize feedback in four major areas: (1) like (positive feedback), (2) impact (potential impact of the app), (3) change (suggestions for improvement), and (4) ideas (ideas for novel app features; Table 5).

**Table 4.** Prototype ratings Individuals rated prototype features using a 5-point Likert scale (1=Loveit, 5=Hateit).

Features	Prototype 1 (n=9)	Prototype 2 (n=7)
Features, mean (SD)		
Education	1.6 (0.7)	Not rated
Self-Assessment quizzes	2.1 (1.2)	Not rated
Goal setting and logs	1.6 (1.0)	Not rated
Trackers (steps/sleep/mood)	1.7 (1.0)	Not rated
Reminders	1.4 (0.7)	Not rated
Community/Social connection	1.4 (0.5)	Not rated
Format, mean (SD)		
Color scheme	2.1 (0.8)	2.1 (1.1)
Layout	2.0 (0.9)	1.1 (0.4)
Text	2.0 (1.0)	1.9 (0.9)
Graphics/Images	1.9 (0.9)	1.9 (0.9)
Likeliness to use app	1.7 (0.9)	1.7 (0.8)

**Table 5.** mHealth app prototype feedback.

Feedback category	App feature/characteristic	Theme	Exemplar quotation
Like	Educational modules Graphics Messages/ Notifications Interface Trackers	Reliable educational information Validation · Self-management Usability · Clinical care; self-management	“I think that every lupus patient should have educational materials that give them important information and insights into how they can manage their fatigue.” “What I like especially, is the person in a wheelchair. Because when you say activities, usually people think of someone who is already standing up and who is able to do something. But, you have a wheelchair and you have exercise. It gives motivation to people that are in the same situation.” “Reminders are great for lupus fog!!! Also great if there’s some kind of encouragement involved in the message.” “It doesn’t feel like it’d be a huge learning gap if someone is going from another health tracker to this one. It doesn’t feel disorienting if they’ve used a health tracker before. This feels exactly the same, and to me that’s a good thing.” “The ability to utilize, or be able to integrate this information, all this data, to show or to report to the physician. How helpful is this going to be for the patient to manage, or to help the doctor understand how active or not active they’ve been or how they’ve been feeling.”
Impact	Trackers	Self-management Self-management Clinical care	“This notion of really checking on your quality of life and your mood is such an important part of it, because I think that’s sort of the underserved aspect or component of this journey.” “This is not only going to help me, it is going to push me to do better, it is going to show me [to recognize] what I need to work on more.” “This is something that is going to help on my doctor visits because my doctor asked me questions like how you’ve been feeling, have you been sleeping normally.”
Impact	Journal	Validation	“Knowing that I have my [app] best friend can be like ‘hello, [name] how are you feeling today.’ Sometimes we just need that.”
Change	Trackers	Usability Gamification Usability	“A lot of it requires patient self-report, patient logging. There’s a lot of effort needed on the patient side.” “It’s just not worthwhile if there’s no real time feedback or incentive for me to continue logging in or putting in my information. It can get tedious.” “I was hoping that it communicated with and was able to pull that data from apple health or fitness.”
Change	Graphics	Validation	“I want to feel connected with some of the images...I’m the type of person who loves to see real people. I want to feel that connection.”
Change	Notifications	Customizability	“People want to be reminded when they want to be reminded. Not that someone reminds them at any given time.”
Ideas	Trackers	Self-management Self-management	“A food log...trying to help people find that correlation to like ‘Oh, I felt bad because I may have eaten a food like a nightshade or something.” “Let’s say you want to upload a video of your activity...I can actually take video of the progress like actually show the physical progress in video format.”
Ideas	Educational Modules	Gamification	“When a module is completed or something you get a badge and there’s emojis coming out. Like you reached this level.”
Ideas	Messages/Notifications	Self-management Self-management Self-management	“Maybe there’s a reminder piece: ‘Hey it’s nine o’clock. Take your medicine.” “A pop-up reminder...don’t forget to pace yourself.” “Little words of affirmation. Something to encourage you.”
Ideas	Community	Social support Social support	“It would be kind of cool to have it all-inclusive to people that have lupus and maybe a parent guardian or friend...where I can invite my mom to be a part of this app.” “If there was some sort of way that...you could friend people, almost like it is its own social network.”

Key themes and insights that emerged from the interviews in each of these 4 areas are summarized below.

- Like: Overall, participants liked the educational modules, graphics, messages and notifications, trackers, and app interface. The educational modules were felt to be useful and easy to understand, while the trackers were recognized as important tools for monitoring symptoms. The graphics were considered motivating, especially for their inclusivity in depicting people in wheelchairs and engaging in diverse activities. The app interface and functionality were perceived to be intuitive.
- Impact: Participants highlighted the trackers and journal features as particularly impactful. They felt that the trackers allowed for self-motivation, reflection, and sharing data with their physicians. The journal feature, which allows individuals to annotate their mood, energy, and activity logs, was felt to be validating and personalized.
- Users identified several key areas for potential change. They felt that the tracking features were burdensome, requiring users to actively log activity and symptoms. They noted this burden could be reduced with integration of the app with preexisting health apps, including Apple Health (Apple). They felt the app could be more engaging and use gamification to reward milestones. While some users found the graphics appealing, as noted in the “like” section, others felt that using real images of people would create a stronger connection. Finally, users desired app customizability, such as the ability to choose which symptoms to track and the option to select if and when to receive notifications.
- Ideas: Participants generated ideas for several novel app functionalities, including dietary trackers or food logs and video logs or journaling. They desired notifications, especially reminders to pace physical activity and take medications, but also messages of encouragement and affirmation. Several cited a need for gamification related to completing educational modules and achieving activity goals. In addition, users wanted expanded social support options, such as “friending” capabilities and live chat within the app, and the ability to invite family members to join the app “community.”

This feedback was used to iteratively refine the prototype. Key updates included the addition of a banner to congratulate users upon completion of educational modules and enhanced app functionality, such as integration with Apple Health or Google Fit to automatically track movement, steps, and sleep data. Of note, several suggested features, including food logs, medication reminders, and a social network/community with live chat, were not incorporated into the mHealth intervention given the scope of our design challenge and focus on physical activity.

The refined prototype was shared with participants in a second round of feedback interviews, which focused on evaluating the format and usability of the app. Ratings of the second prototype were generally favorable (Table 4). The following changes were made based on this round of interviews: (1) font size was increased to improve readability and accessibility, (2) the mood question was clarified, and (3) a graphic was added to the activity log to enhance representation. The screenshots of early and final prototypes of the app are illustrated in Figures S1 and S2 in Multimedia Appendix 3.

A recurring theme in feedback interviews was the user desire for app-based notifications, including reminders and educational and motivational messaging around physical activity and fatigue management. To meet this need, we undertook the creation of a digital messaging feature as part of the app. A literature search was performed to find existing messaging libraries that promoted physical activity and/or addressed fatigue management and were grounded in behavior change theory. A published text messaging library created to support a diabetes prevention program was identified [64,65]. These messages were developed to promote healthy diet and physical activity and were mapped to specific behavior change techniques from the Behavior Change Wheel framework, a comprehensive model that synthesizes multiple behavior change theories [66]. This library was selected as the foundation for developing messages for our mHealth intervention because of its focus on promoting physical activity and rigorous incorporation of behavior change theory.

The 124 messages in the published library were reviewed, and messages specific to the diabetes program or content related to diet were removed as these were not relevant for our intervention. The remaining 69 messages were adapted to be more specific to people with SLE. Forty-nine new messages about physical activity were generated based on feedback from the first round of interviews and the educational modules developed for the mHealth intervention. Message content included positive self-talk, reminders to pace yourself, and reminders to hydrate. The newly developed physical activity messages were mapped to relevant behavior change theories by 2 investigators working independently (AD and PC), and coding was then compared until a final coding was assigned for each message. Codes were reviewed by 2 other investigators (SK and SF), and messages were edited to improve autonomy-supportive language in keeping with a central concept of SDT. Thirty new messages related to fatigue were also created and included testimonials from individuals living with lupus, reminders to gauge energy levels, and strategies to manage fatigue. All messages were revised to ensure an eighth-grade or lower reading level using Readability.io and were limited to 160 characters to fit within a standard SMS text message.

Interview participants were then invited to review the messages and provide feedback in a third round of interviews. Ten individuals participated and rated the messages highly, with an average message rating of 1.3 out of 5 (where 1 is highest). Four messages were removed from the library based on low ratings by participants. Interviewees shared open-ended comments, which were used to revise the messages. Twenty-seven messages were modified to minimize “lupus warrior” phrasing, which received mixed feedback, and to remove “work-out,” “exercise,” “strength-based,” or “aerobic” as these terms were felt to be more intimidating than “movement” or “physical activity.” Several messages were edited to use more autonomy-supportive language. Eleven new messages were generated based on feedback about content missing from the library, including “lupus life hacks,” managing pain, and evolving while living with lupus. The complete messaging library consisted of 154 messages (Table S1 in Multimedia Appendix 4).

## Discussion

### Principal Findings

Our study used an HCD process to develop a novel mHealth app, grounded in behavior change theory, for self-management of fatigue in SLE. We describe the first 2 phases of development: inspiration and ideation. In the inspiration phase, we identified key user needs, including high-quality educational resources, symptom and activity tracking features, social connection, and reminders. In the ideation phase, we created an app prototype using an iterative process informed by feedback from people with SLE at every stage of the design process. One key point of iteration was the user desire for a wide range of notifications and messaging. Based on this feedback, we generated a novel messaging library anchored in behavior change techniques for use with the mHealth app. Using this approach, we were able to create an mHealth app tailored specifically to stakeholder needs that users reported they were highly likely to use.

Our app offers a unique tool for the self-management of fatigue in SLE. Prior apps designed for this patient population often lacked reliable educational information and had limited functionality, with little involvement from people with SLE in the design process [44,67]. Existing mHealth apps for the self-management of SLE have shown potential to improve quality of life metrics but could benefit from a stronger HCD and a more rigorous approach to behavior change theory [68]. By applying an HCD approach and engaging stakeholders throughout development, we identified essential mHealth functions valued by individuals with SLE. Participants in our study expressed a need for symptom trackers, specifically for fatigue, pain, sleep, and physical function, which aligns with previous findings [69]. In addition, we identified key priorities for mHealth tools among those with SLE, including social connection, accessibility and inclusion, customizability, and integration with existing digital health tools for disease self-management. These aspects have received limited attention in the literature to date.

mHealth apps have been successfully developed for the self-management of various chronic conditions, including chronic pain, diabetes, rheumatoid arthritis, and chronic lung disease [70-73]. Many of these apps have incorporated symptom tracking, medication reminders, lifestyle monitoring, and education, while also integrating behavior change theory and stakeholder feedback into their design [74]. However, to our knowledge, this process has not yet been systematically applied to mHealth interventions for SLE.

Our mHealth app, with its symptom and physical activity tracking and evidence-based education, shares features with these previously developed self-management apps. However, it was specifically designed to address the unique needs of individuals with SLE, particularly in the context of fatigue management. While our prototype includes core features such as symptom tracking, lifestyle monitoring through physical activity tracking, and high-quality educational modules, we were unable to incorporate medication tracking, reminders, or food logs due to design constraints. Notably, these features were also identified as important by participants and represent potential areas for future development.

Through its grounding in behavior change theory and its emphasis on user-centered design, our mHealth app fills a critical gap in existing digital health tools for SLE. Future research is planned to evaluate the mHealth app in the third and final stage of the HCD process, implementation. We plan to pilot test the usability and effectiveness of the app in a clinical trial of a peer coaching intervention for SLE-related fatigue. Findings from this study will inform further refinements and implementation of the app.

Limitations of this study included a small sample size and design constraints. Each round of interviews had a small sample size, which could have biased the results of this study. While participants were purposively sampled based on demographics and disease characteristics to represent a variety of perspectives, it is possible that the needs identified in the focus groups and interviews do not represent those of the entire population of people with SLE. In addition, the app development was restricted in scope by our design challenge to address fatigue in SLE and by budgetary constraints. This limited our ability to create an mHealth app that addressed every feature requested by interview participants.

### Conclusion

In conclusion, this study presents a structured methodology for developing an mHealth app to support fatigue self-management in individuals with SLE. By using an HCD approach and integrating behavior change theory, we created a tool that directly addresses the needs of people with SLE—a group for whom digital health technologies have been limited. Our iterative design process, which actively engaged end users, demonstrates the feasibility of applying HCD in this setting and underscores the value of user involvement in enhancing adoption and usability. This approach offers a rigorous framework for mHealth app development that can be adapted to further expand digital health solutions for the self-management of SLE and other chronic conditions.

## Supplementary material

10.2196/75399 Multimedia Appendix 1 Focus group guide.

10.2196/75399 Multimedia Appendix 2 Interview guide.

10.2196/75399 Multimedia Appendix 3 Prototype screenshots.

10.2196/75399 Multimedia Appendix 4 App messages.

## Abbreviations

HCD: human-centered design
*ICD-10*: *International Classification of Diseases, Tenth Revision*
*ICD-9*: *International Classification of Diseases, Ninth Revision*
mHealth: mobile health
REDCap: Research Electronic Data Capture
SCT: social cognitive theory
SDT: self-determination theory
SLE: systemic lupus erythematosus
