# Supplementary material for: Development of a Theory-Based mHealth App for Fatigue Management in Lupus: Human-Centered Design Approach
Source: JMIR Form Res. 2025 Aug 26;9:e75399. doi: 10.2196/75399 (PMC12380406; doi:10.2196/75399)
Supplement: Multimedia Appendix 1 [file formative-v9-e75399-s001.docx]

**Focus Group Guide**

**[Total Running Time: 35 min]**

**Introduction to the Focus Group [5 min]:**

***Welcome***

Hello and welcome. Thank you for taking the time to join our discussion today. My name is [ ] and I will be asking you questions and moderating the group.

***Overview of the Topic***

I am going to be reading directly from this script to make sure we don’t miss anything. We are gathering you together here today to learn more about your experience living with lupus and fatigue, and resources that might help you manage this symptom.

***Ground Rules***

We have a few ground rules for this session. There are no right or wrong answers. We expect that you will have differing points of view. Feel free to share your point of view even if it differs from what others have said. Our purpose is not to develop a consensus or to all agree, but is to hear everyone’s perspective. Please remember to show respect for what others may have to say. You may want to talk about other things related to your lupus and while these are very important, my job is to keep us focused on the topic of lupus and fatigue. If other issues are raised, please remind me, and we will see how we can address them later. Also, in our roles here today we cannot offer any medical advice.

We’re recording the session because we don’t want to miss any of your comments. Your comments are confidential. No names will be included in any reports. If you say something that could identify you – like the name of your doctor or a family member’s name – we take that out when we create a written version of this conversation.

If you have a cell phone or pager please check to make sure it is turned off now.”

[*Moderator note*: PAUSE to allow time to turn off phones]

**Opening Question: [2 min]**

Now I am going to ask you a series of questions, starting with very general to more specific. So let’s begin. First, it would be nice to go around the room to learn a little about each other.

- Tell us your name and how long you have had lupus. [*Moderator note*: go around table]

**Introductory Question: [5 min]**

- What is the single most challenging way that lupus affects your day to day life? I know it may be difficult to choose one, but we will have an opportunity to discuss these challenges later. [*Moderator note*: go around table]

**Managing Fatigue in Lupus [20 min]**

Let’s talk about a symptom that some of your mentioned that is very common in lupus: fatigue.

1. We know that fatigue is an important symptom that both you and your doctors struggle with. We are planning to develop a tool (i.e. a mobile health application) to help you manage your fatigue. What are some of the features you would like to see in this tool? [5 min]

[*Moderator note:* Examples could include educational information, tracking tools, peer support, connecting with an expert]

1. What symptoms or activities would you want to track in order to manage your fatigue? [5 min]

2. What information or strategies would help you manage your fatigue? [10 min]

1. Probe Questions:
2. For example, would you like information about the causes of fatigue in lupus?
3. Would you like information about how the following affect fatigue:
   1. Physical activity
   2. Diet/Nutrition
   3. Sleep hygiene (good sleep habits)
   4. Complementary or integrative health strategies(ex. mindfulness)

**Ending Question/Closing:** **[3 min]**

- - - - 1. Before we end, looking back at our discussion is there anything we missed or anything else you would like to share with us?

Thank you all for sharing your thoughts and ideas.
