# Supplementary material for: Development of a Theory-Based mHealth App for Fatigue Management in Lupus: Human-Centered Design Approach
Source: JMIR Form Res. 2025 Aug 26;9:e75399. doi: 10.2196/75399 (PMC12380406; doi:10.2196/75399)
Supplement: Multimedia Appendix 2 [file formative-v9-e75399-s002.docx]

**Semi-Structured Interview Guide**

**Introduction:**

Thank you for joining me today. My name is [interviewer’s name] and I am a [role on the study] with the Fatigue in Lupus study. As you know, we are developing a program to help people with lupus manage fatigue. We are working together with patients to make sure that the program meets their needs. I appreciate you taking the time to share your thoughts with me today.

Our conversation will take about 30 minutes. Anything we talk about is confidential, meaning that it is strictly being gathered to help us design this future program. Your personal information will not be linked to your comments.

There are no right or wrong answers. Please feel free to express yourself openly. Anything you have to say – positive and negative – will be very helpful. With your permission, I would like to record the session to make sure there is an accurate record of it so that the study team can go back to review important details. We will use the recording to create a written transcript of the conversation and take out any identifying information, like your name or the name of your doctor. Do you have any questions before we get started?

**Warm Up: Get to Know the Patient/Stakeholder**

1. Tell me about yourself.

Probe: Where are you from? What’s your favorite food?

2. Tell me about your journey with lupus.

1. When were you diagnosed with lupus?
2. What are some of the ways in which lupus has affected your life?

**Topic 1- Understand the Patient/Stakeholder’s Experience with Fatigue and Physical Activity**

1. What does fatigue mean to you as someone living with lupus?

2. What strategies have you tried to manage your fatigue?

3. Tell me about what physical activity or movement means to you in your day-to-day life.

4. How has this changed since your lupus diagnosis?

**Topic 2- Prototype**

Now let’s look at a theoretical mobile health application (or “app”) to help people with lupus manage their fatigue. I will walk you through screenshots of the app and highlight the different features. Please feel free to ask me any questions as we go through the screenshots. After we look at all the screenshots we will talk about your impressions.

*[Share screenshots via “share screen” and do “playthrough” during interview. Highlight different features of app including educational content & quizzes, goal setting, activity logs, trackers for steps, sleep, mood, surveys, and peer coach].*

1. Please share with us your overall thoughts about this app.

Probes:

- What do you like about the app?

- What would you change?

- What is missing?

Consider:

- Features: educational modules, quizzes, goal setting and activity logs, trackers (physical activity, surveys, mood, steps, sleep), connect with coach, reminders

- Home page (how are you feeling today w/ emojis)

- Color scheme, layout, text/font, graphics/icons

2. Prototype Survey *[Share the Prototype Survey in real time via REDCap link, survey questions below for reference]:*

- Please rate the app features. [1= Love them – 5= Hate them], followed by the question: “Please share your reason for this rating.” [Free text])

- Educational modules
- Quizzes
- Goal setting/activity logs
- Trackers (steps, sleep, mood)
- Reminders
- Connect with peer coach

- Please rate the app interface. [1= Love it – 5= Hate it], followed by the question: “Please share your reason for this rating.” [Free text])

- Color scheme
- Layout
- Text (font style and size)
- Graphics/images

- How likely are you to use this app? [1=very likely to 5=very unlikely]

- Why or why not? [Free text]

3. Tell me about your experience, if any, with fitness trackers like FitBit or Apple Watch.

4. What are your thoughts about using trackers (for steps or sleep) as part of a program to manage fatigue?

5. We are planning to set up some kind of support system within the app. How would you prefer to connect with others with lupus for support or community while using this app?

Probes:

- Online community forum (like reddit or facebook group) with other app users and moderators where you can share your experience and challenges
  - What are your concerns? [Comfort sharing information knowing that precautions to protect participants privacy would be would be taken but could not be guaranteed?]
- Online group meeting with peer coach: app users and peer coach meet to share experiences and challenges

**Wrap Up**

Thank you for being open and sharing your experiences with me. It will really help our team understand how we can better support people with lupus.

[SUMMARIZE.] Does that accurately reflect what you told me? Is there anything else you would like to follow-up on or mention?

Do you have any questions for me about anything we’ve talked about today or the research we’re doing?

Again, thank you for your time.
