# Supplementary material for: Development of a Theory-Based mHealth App for Fatigue Management in Lupus: Human-Centered Design Approach
Source: JMIR Form Res. 2025 Aug 26;9:e75399. doi: 10.2196/75399 (PMC12380406; doi:10.2196/75399)
Supplement: Multimedia Appendix 3 [file formative-v9-e75399-s003.docx]

Supplemental Figure 1. mHealth App Prototype Screenshots


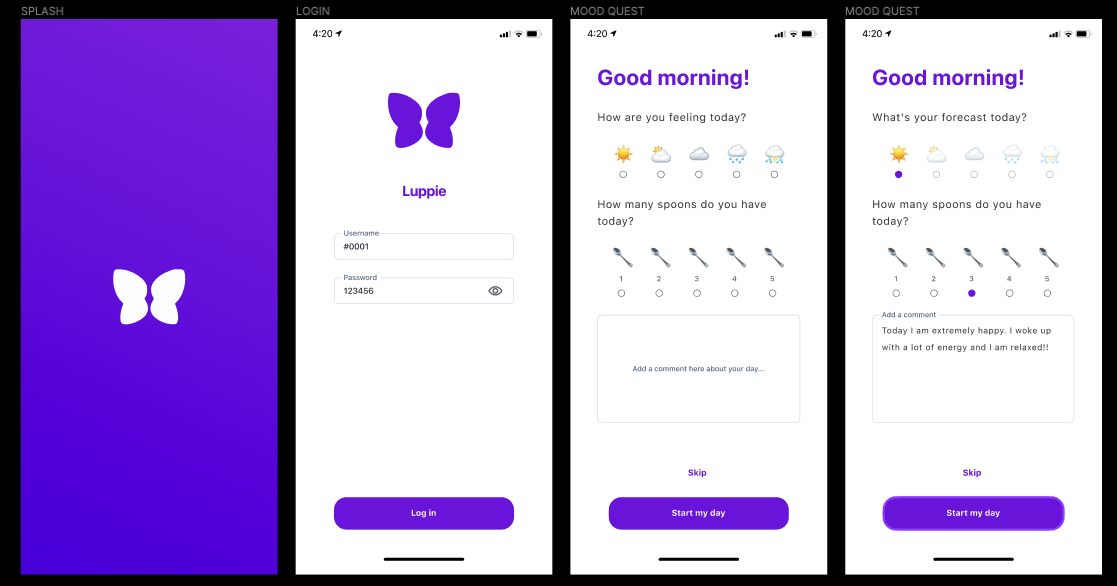

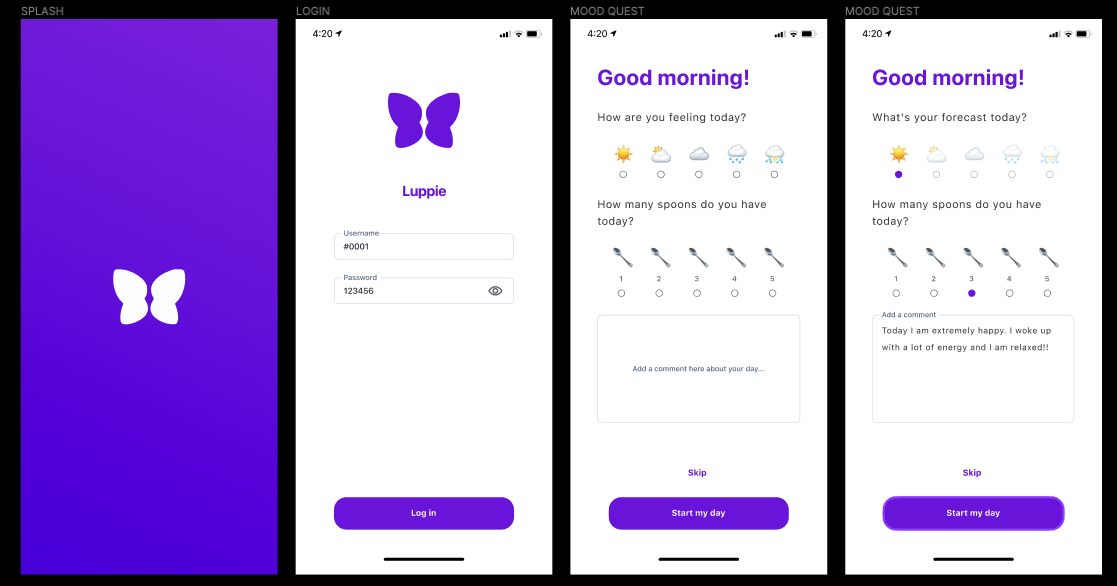

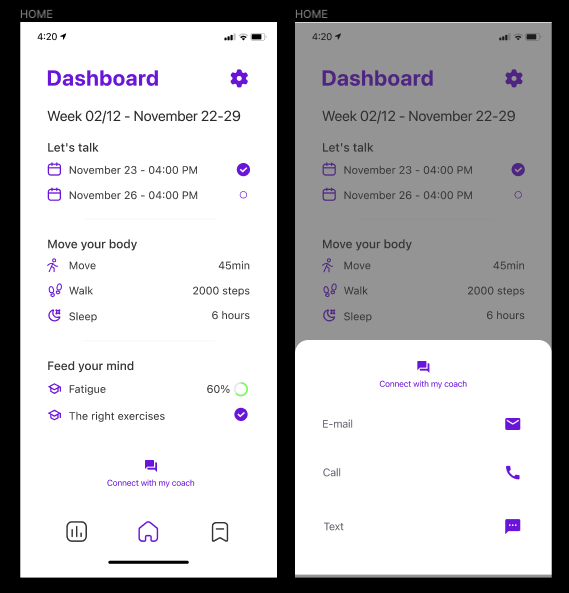


a) Landing page, mood and energy log, and dashboard


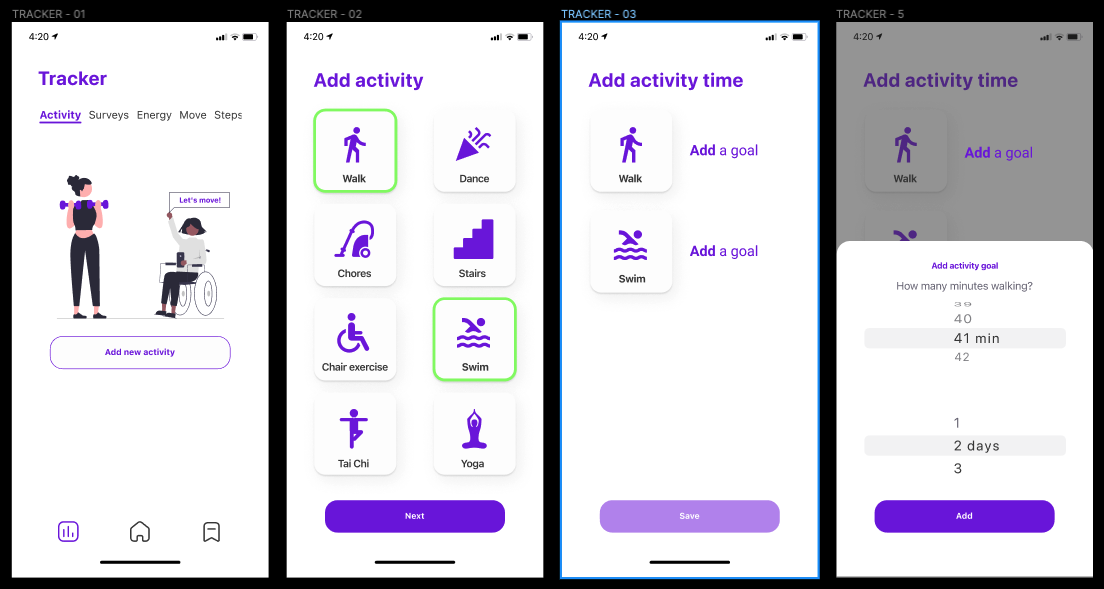


b) Activity tracker


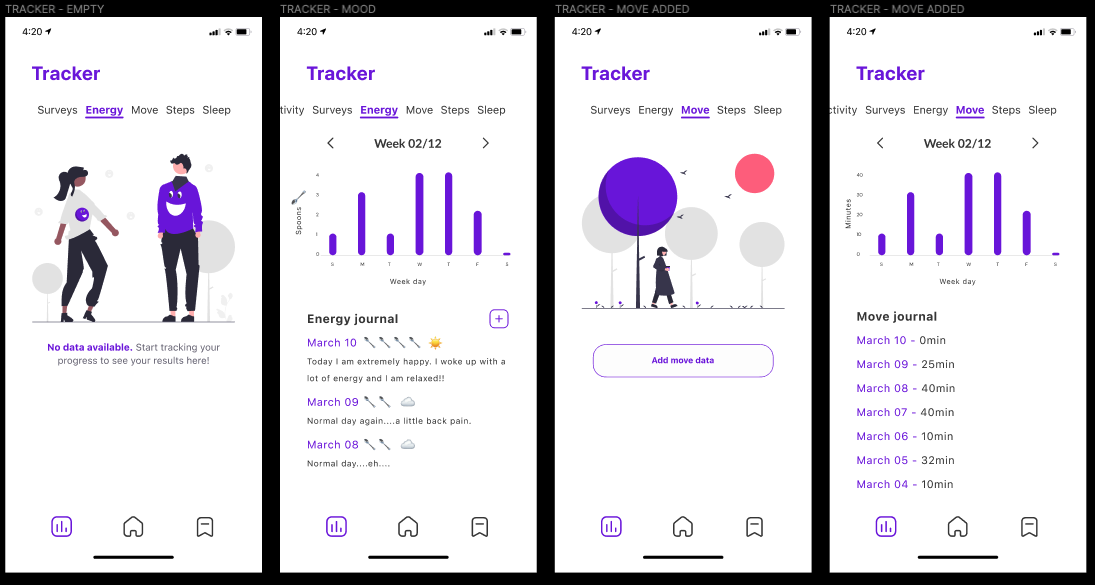

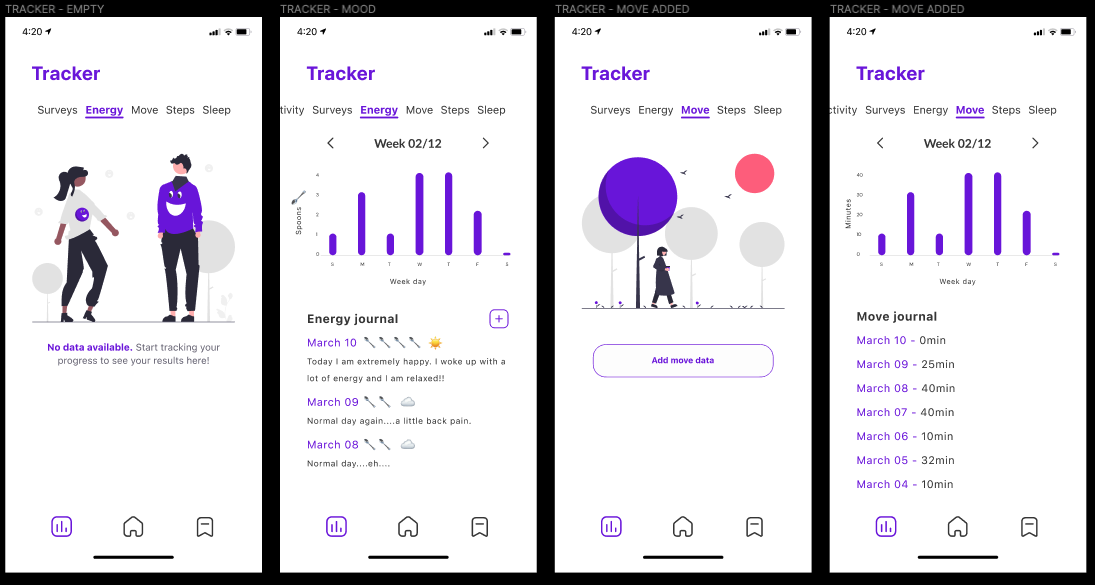

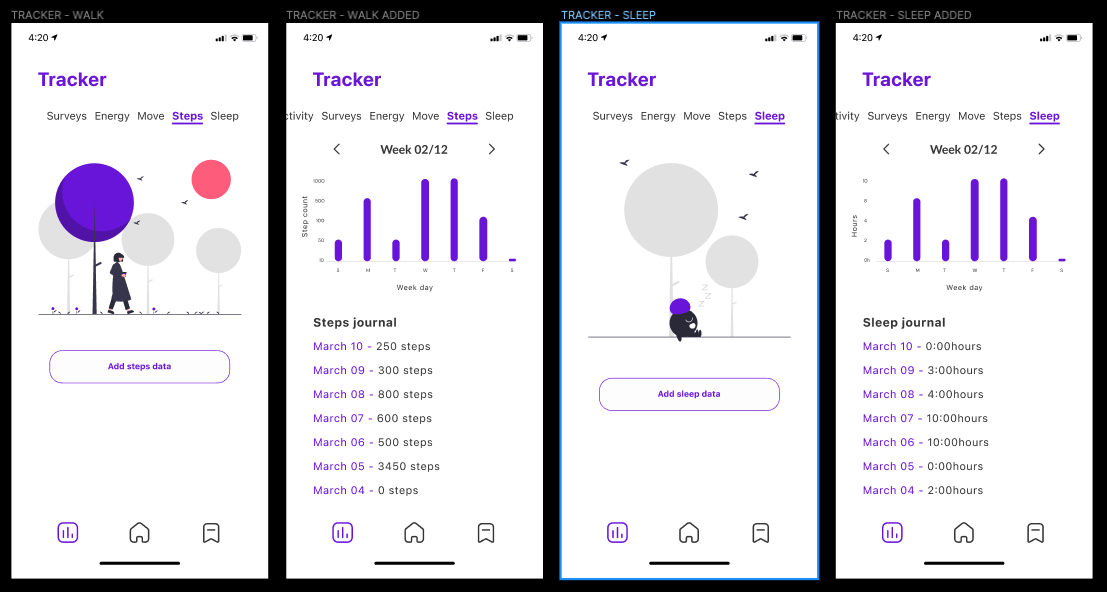


c) Energy, move, and step trackers


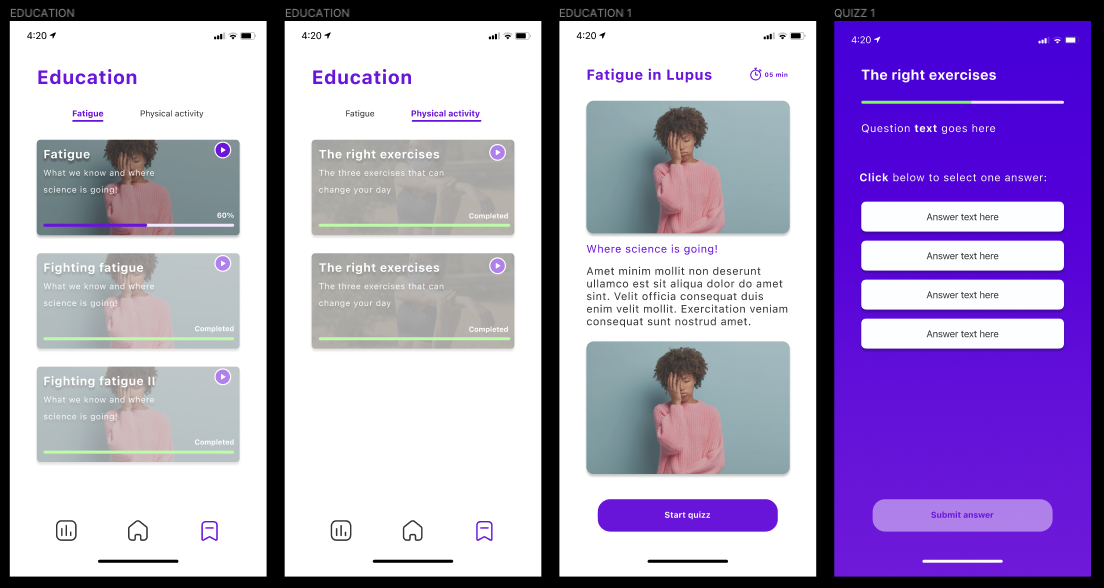


d) Education


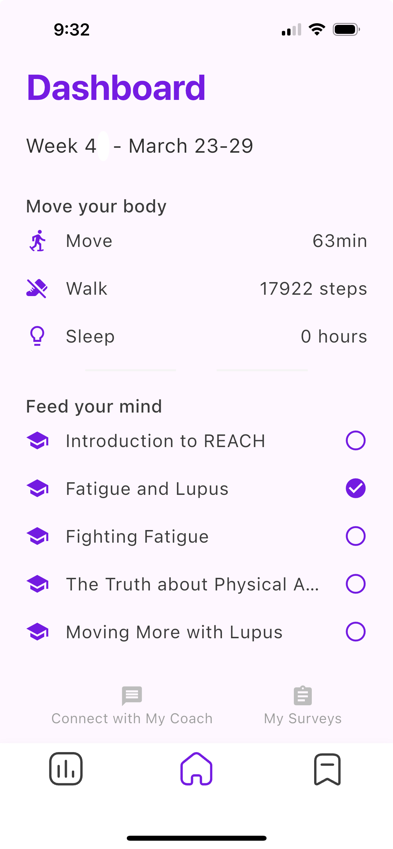

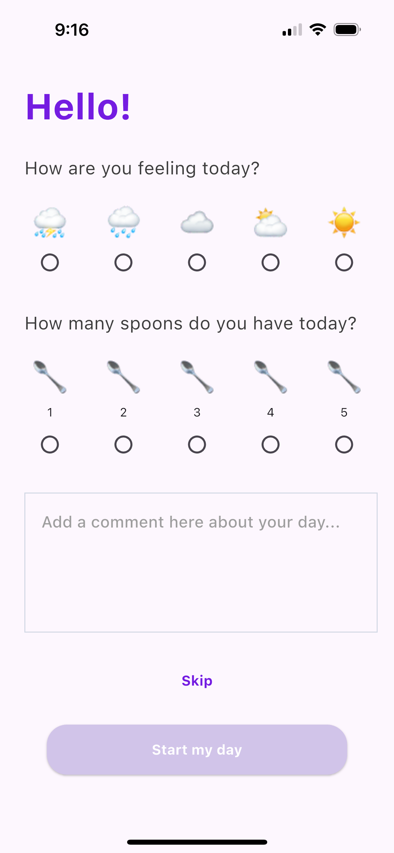
Supplemental Figure 2. Final mHealth app Screenshots


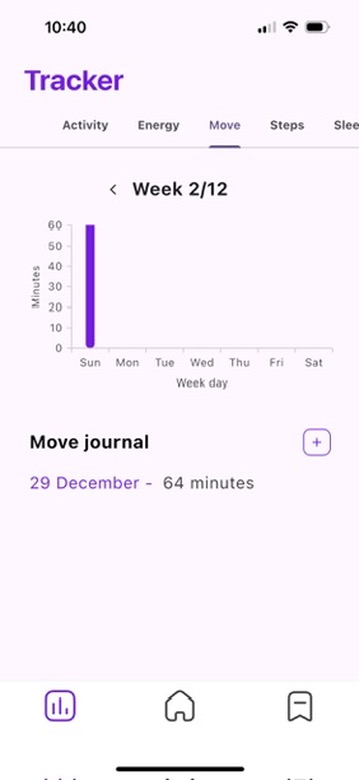

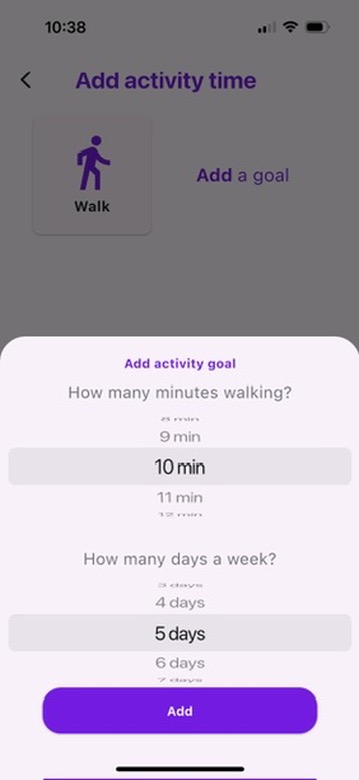

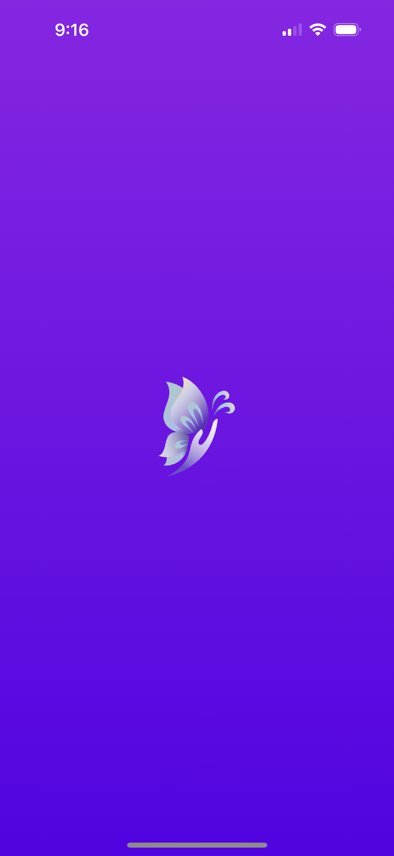
a) Landing page, mood and energy logs, and dashboard

b) Activity tracker


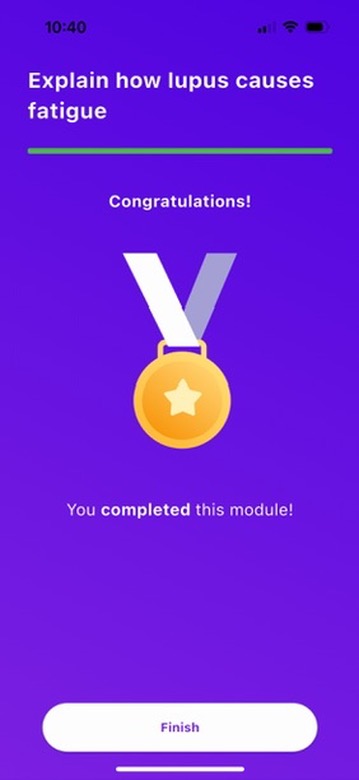

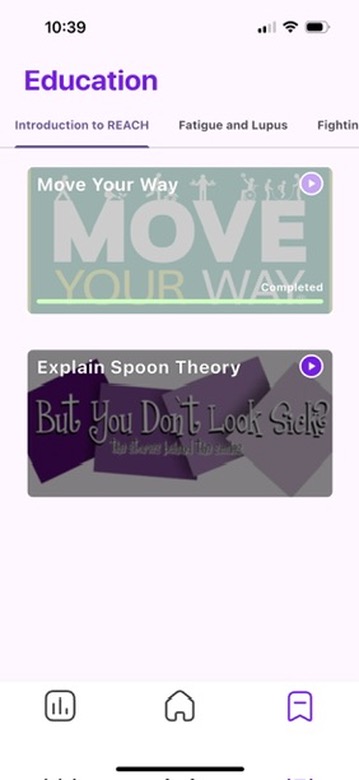

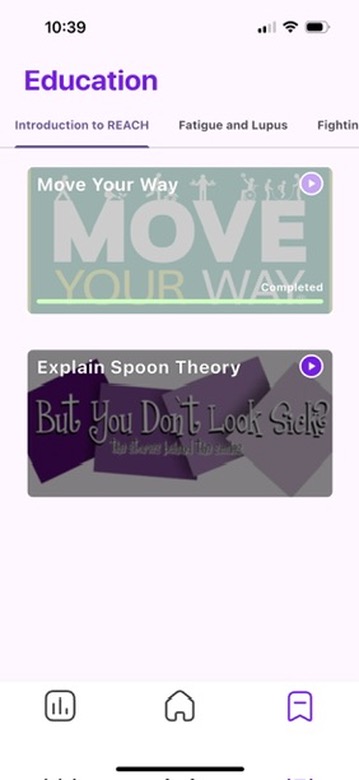

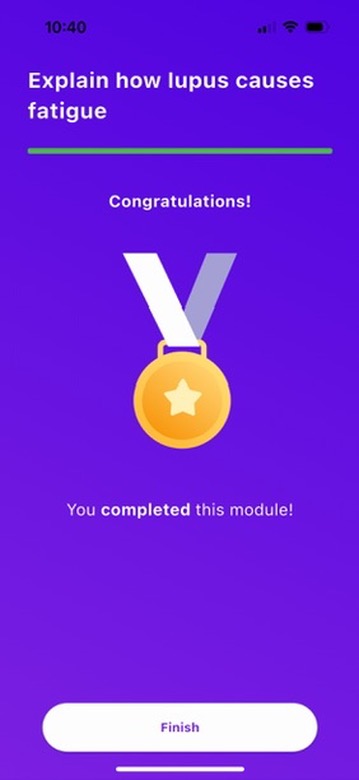
c) Educational modules and completion award
