# Supplementary material for: Development of a Theory-Based mHealth App for Fatigue Management in Lupus: Human-Centered Design Approach
Source: JMIR Form Res. 2025 Aug 26;9:e75399. doi: 10.2196/75399 (PMC12380406; doi:10.2196/75399)
Supplement: Multimedia Appendix 4 [file formative-v9-e75399-s004.docx]

Supplemental Table 1. Final app message library with behavior change techniques. Messages 1 to 48 were developed de novo to pertain to physical activity and SLE. Messages 49 to 85 were developed de novo to pertain to fatigue and SLE. Messages 86 to 154 were adapted from a preexisting library (M. MacPherson et al., 2021; M. M. MacPherson et al., 2021). Messages promoting behavior change related to physical activity were coded using behavior change technique taxonomy v1 (Michie et al., 2013).

|  | **Message** | **Behavior Change Technique (Behaviour change technique number)** |
| --- | --- | --- |
| 1 | Anything that gets your body moving counts toward your movement goal. Household chores and walking are great ways to get in some physical activity. | Instruction on how to perform the behavior (4.1) |
| 2 | One way to think about moving more is by spending less time sitting still. Moving your arms and legs while sitting counts! | Behavior substitution (8.2) |
| 3 | It can be tough to get moving with lupus. It is important to always listen to your body and slow down if you need to. | Monitoring of emotional consequences (5.4) |
| 4 | Movement is safe for people with lupus. Studies show that physical activity does not cause lupus flares. | Information about health consequences (5.1) |
| 5 | Low impact activities, like walking, have less risk of injury. Consider moving slowly and build up movement over time. | Instruction on how to perform the behavior (4.1); Graded tasks (8.7) |
| 6 | Movement has lots of benefits for people with lupus. Getting moving can help with fatigue, mood, and pain. The benefits start to add up with just 5 minutes of activity each day. | Information about health consequences (5.1) |
| 7 | Benefits of movement include better sleep, better mood, and better focus. The benefits start to add up with 5 minutes of activity each day. | Information about health consequences (5.1); Graded tasks (8.7) |
| 8 | When making a goal, try to keep it simple and specific. Consider walking for 10 minutes a day or trying a short exercise video. | Goal setting (behavior) (1.1) |
| 9 | Remember to pace yourself. Consider adding breaks to your movement to rest when you need it. | Self-monitoring of behavior (2.3); Monitoring of emotional consequences (5.4) |
| 10 | If movement hasn't been part of your routine before, start slowly. You can begin with 5 minutes of an activity and work your way up. | Graded tasks (8.7) |
| 11 | Always listen to your body. If you feel stiff or sore, or if you feel your lupus flaring, take a break. | Self-monitoring of behavior (2.3); Monitoring of emotional consequences (5.4) |
| 12 | Getting moving can be scary at first. This is a normal feeling! Consider trying a workout at home or going for a walk with a friend. | Problem solving (1.2); Restructuring the physical environment (12.1) |
| 13 | Reflect on your personal reasons for getting moving. This could include wanting to feel less tired or trying out new activities with friends. | Review outcome goals (1.7) |
| 14 | Having fun while moving helps you stick to your goals. Choose activities you like to do, like dancing or walking. | Monitoring of emotional consequences (5.4); Information about emotional consequences (5.5) |
| 15 | Getting moving might make you feel a little tired or make your muscles sore. But, physical activity should not cause pain or exhaustion. | Information about health consequences (5.1) |
| 16 | Try warming up before moving and stretching once you've finished your activity. These practices keep your body safe and help your muscles and joints feel better. | Instruction on how to perform the behavior (4.1); Information about health consequences (5.1) |
| 17 | It is normal to feel nervous about becoming more active. Consider setting a small goal for movement to build some confidence. | Information about emotional consequences (5.6); Monitoring of emotional consequences (5.4) |
| 18 | “There are days when lupus fatigue and aches can be overpowering, but if I stretch for 3 minutes at a time, it can make all the difference.” -Faye C., Lupus Warrior | Credible source (9.1) |
| 19 | Meeting your goals won't always be easy. Try journaling or talking to your support network if you're feeling discouraged. | Information about emotional consequences (5.6); Self-monitoring of outcome of behavior (2.4); Reduce negative emotions (11.2); Social support (emotional) (3.3) |
| 20 | There are some weeks when you might feel more tired. Remember that it’s okay to listen to your body and take a break when needed. | Monitoring of emotional consequences (5.4); Information about emotional consequences (5.5) |
| 21 | Movement may make your muscles sore. Stretching after movement and using a heating pad can help loosen them up. | Instruction on how to perform the behavior (4.1) |
| 22 | If you don't meet your goal, try reflecting on the challenges you faced. For example, if you were tired in the evening, add movement to your morning routine. | Discrepancy between current behavior and goal (1.6); Problem solving (1.2) |
| 23 | Remember to adjust your plan as needed. See how next week goes and stay with it! | Review behavior goal (1.5) |
| 24 | Reward yourself for your success. Try a relaxing bubble bath or anything you enjoy after achieving your weekly movement goal. Think about what will motivate you! | Self-reward (10.9) |
| 25 | Lupus warriors can develop rashes and skin damage from sunlight. Using sunscreen and dressing for the weather can help protect your skin. | Information about health consequences (5.1); Instruction on how to perform behavior (4.1) |
| 26 | Different temperatures can cause discomfort for lupus warriors. Consider working out inside on days when the weather isn't right for you. | Information about health consequences (5.1) |
| 27 | Staying hydrated helps keep lupus warriors strong. Fluids keep your kidneys healthy and help you cool down. | Instruction on how to perform the behavior (4.1); Information about health consequences (5.1) |
| 28 | Some people find it hard to set movement goals if they haven't been active before. [You can click here](https://www.arthritis.org/health-wellness/healthy-living/physical-activity/walking/building-a-walking-workout) to get some ideas for building a walking work out. | Instruction on how to perform the behavior (4.1); Goal setting (1.1) |
| 29 | Including strength-based activities in your routine can help counter the side effects of steroids. These activities help build bone and muscle. | Information about health consequences (5.1) |
| 30 | Some people find it easier to start moving in a familiar place. Consider trying to get active in your home or backyard. | Restructuring the physical environment (12.1); Problem solving (1.2) |
| 31 | Dress for success! Consider what clothes and shoes will help you move with ease while you are active. | Body changes (12.6) |
| 32 | Consider finding a role model as you start moving. Ask someone who's reaching their activity goals for their story and advice. | 3.2 and 3.3 Social support (practical and emotional) (3.2 and 3.3) |
| 33 | It can be hard to figure out what kind of movement is right for you. Consider checking out [Move Your Way](https://health.gov/moveyourway) for some ideas! | Instruction on how to perform the behavior (4.1) |
| 34 | It can be hard to get moving if it’s not part of your daily routine. Link movement to something in your routine (e.g. march in place as you brush your teeth). | Habit formation (8.3) |
| 35 | As you get moving, it's important to pace yourself.  Give yourself permission to go at your own speed. | Monitoring of emotional consequences (5.4); Self talk (15.4) |
| 36 | Check out the [Arthritis Foundation](https://www.arthritis.org/health-wellness/healthy-living/physical-activity/getting-started/your-exercise-solution) for ideas for safe movements and gentle exercises for different joints! | Instruction on how to perform the behavior (4.1) |
| 37 | Moving reduces joint stiffness and pain in people with lupus. | Information about health consequences (5.1) |
| 38 | Benefits of movement include better mood, improved sleep, and brain health. | Information about health consequences (5.1) |
| 39 | Keep up the great work wearing your fitness tracker! Remember, staying active is the best medicine for fatigue! | Information about health consequences (5.1) |
| 40 | Some benefits of physical activity happen right away. Others take more time, like improved bone health and a lower risk of heart conditions. | Information about health consequences (5.1) |
| 41 | As you get moving, check in with your body. Talk to your doctor if you feel any new pain or discomfort. | Self-monitoring of outcomes of behavior (2.4) |
| 42 | If you’re not feeling confident, try setting a smaller goal and show yourself what you can do. | Graded tasks (8.7); Self-monitoring of outcome of behavior (2.4); Reduce negative emotions (11.2) |
| 43 | “Be kind to yourself. Warriors have tough days too.”- Chris, Lupus Warrior | Reduce negative emotions (11.2); Self talk (15.4); Verbal persuasion about capability (15.1) |
| 44 | Progress, not perfection. Remember: A lapse only turns into a relapse when you stop trying. | Framing/reframing (13.2) |
| 45 | One way to deal with the fear of getting started is to start small. Set a short time limit for your movement and check in with your body and mind. | Instruction on how to perform the behavior (4.1); Monitoring of emotional consequences (5.4) |
| 46 | Getting moving can be stressful. Try writing down the emotions you feel before and after working out. You may find that movement helps *relieve* stress. | Monitoring of emotional consequences (5.4) |
| 47 | On days when you don’t feel great, consider adding in just 5 minutes of movement, whatever that looks like to you. | Graded tasks (8.7) |
| 48 | Moving more is most challenging in the beginning. It will get easier with time! | Information about health consequences (5.1) |

**Fatigue Messages**

| **Message Number** | **Message** |
| --- | --- |
| 49 | You are not alone in your fight against lupus fatigue. You can use your REACH app to help you monitor changes in your energy during the week. |
| 50 | Lupus fatigue can make it hard to get through your day.  Listen to your body and consider taking a break (or a nap) if you are too tired to concentrate or move. |
| 51 | Sleep trouble, lack of movement, and stress can all add to your fatigue. Check out the REACH app to learn about what could be making you tired. |
| 52 | What you eat might affect your energy levels. Consider tracking how different foods affect you. |
| 53 | Moving your body is one of the best ways to tackle lupus fatigue. Walking, yoga, and doing chores all can help. There is no one best way to be active! |
| 54 | Being present with your thoughts can help with lupus fatigue. What are some ways you can add mindfulness to your routine? |
| 55 | Low sugar and low-calorie diets may help lupus fatigue. [Click here to learn more about ways that different foods can affect lupus.](https://www.lupus.org/resources/diet-and-nutrition-with-lupus) |
| 56 | Caffeine can affect your quality of sleep. Consider limiting caffeine to the morning to help prevent it from affecting your sleep. |
| 57 | Acupuncture is an option that may be helpful for some people’s fatigue. Consider whether this therapy might be a good option for you. |
| 58 | Managing fatigue is a daily challenge. You can start by planning each day based on your energy levels. If you have low energy, choose the most important tasks. |
| 59 | You might feel more tired some days than others. That is okay! Give yourself permission to take a break or a nap during the day. |
| 60 | Listening to your body is an important way to manage your fatigue. You will restore your energy levels by taking breaks when you need them. |
| 61 | “I’ve learned to be my own best friend by listening to my body There’s days when I feel my body telling me “No, stay.” So, I rest”-Maria H., Lupus Warrior |
| 62 | Be kind to yourself. Remind yourself that your fatigue is not your fault. |
| 63 | Feeling tired does not mean that you are lazy. It’s okay to listen to your body and take a break. |
| 64 | It is okay to ask for help. You can ask a friend to pick up groceries on days you are tired and need a little extra help. |
| 65 | Sharing your experience with lupus with your loved ones can help them support you in your journey. |
| 66 | Try sharing your experience with lupus with your loved ones. Even if they don't fully understand, sharing can help you feel supported in your journey. |
| 67 | Living with lupus can be stressful. Deep breathing exercises or physical activity can help you relax. [Click here to learn more about these types of exercises.](https://palsforhealth.com/Pals/Article/Deep%20Breathing) |
| 68 | One way to de-stress is to try muscle relaxation. This helps you to focus and release tension in your body. [Click here to learn more about muscle relaxation.](https://www.palsforhealth.com/Pals/Article/Muscle%20Relaxation) |
| 69 | Practicing mindfulness can help you deal with the stress of living with lupus. It can help you to be more aware of the present. [Click here to learn more!](https://www.palsforhealth.com/Pals/Article/What%20is%20mindfulness) |
| 70 | “Taking a few moments to breathe deeply and reset helps. On days I just can’t kick fatigue, playing upbeat ‘80s music does the trick.”-Faye C., Lupus Warrior. |
| 71 | Your doctor can help you manage lupus fatigue. Consider asking if your lupus is well controlled. You can ask about other factors that might add to your fatigue. |
| 72 | As you add movement to your day, talk to your doctor. Ask about movements that are safe and if there are activities you should avoid. |
| 73 | Practicing good sleep habits can help you wake up feeling well rested. [Click here for resources on sleeping better when you have lupus](https://www.lupus.org/resources/ways-to-get-better-sleep-when-you-have-lupus%22%20/l%20%22:~:text=A%20calm%20and%20relaxing%20environment,mattress%2C%20pillow%2C%20and%20bedding.). |
| 74 | Water can rejuvenate you if you are feeling tired. What are some ways you can remind yourself to drink water throughout the day? |
| 75 | Staying hydrated can help keep up your energy levels. Consider carrying a water bottle, so you remember to hydrate. |
| 76 | Check in on your spoons today. How many spoons do you have left? [You can learn about the story behind spoon theory here.](https://cdn.totalcomputersusa.com/butyoudontlooksick.com/uploads/2010/02/BYDLS-TheSpoonTheory.pdf) |
| 77 | Do you have enough spoons to do all your tasks for the day? If not, consider picking the most important tasks and leave the rest for another time. |
| 78 | Lupus Lifehack: “Consider ways you can save your energy. Try using disposable plates when you're too tired to do the dishes.”- Nadine L., Lupus Warrior |
| 79 | Try scheduling one day a week to relax. This will help you recover and save energy for the rest of the week. |
| 80 | Remember: You are more than your lupus, and you are doing your best. |
| 81 | Asking for help can be hard. It is okay to lean on those who love you when you need it. |
| 82 | Medications for lupus can help with your fatigue. Are you taking your medications regularly? |
| 83 | You might have more pain on some days than others. Listen to your body and take a break when you need to. |
| 84 | You might have a hard time doing some of the activities you enjoyed before lupus. Consider starting small and try to build back some of your hobbies. |
| 85 | “When I was first diagnosed, I felt like I was in a cocoon. But, like a butterfly, I got my wings. There are stages. It gets better.”- Maria H., Lupus Warrior. |

| **Message Number** | **Message** | **Behaviour Change Techniques (Behaviour change technique number)** |
| --- | --- | --- |
| 86 | Your behavior is an example to those around you. When people see the effort you are putting into being more active, you're likely to inspire others! | Identification of self as role model (13.1) |
| 87 | It's the small steps that lead to big changes in your lifestyle. Every step you take this week is a step towards a more energetic you. | Social support (unspecified) (3.1); Verbal persuasion about capabilities (15.1) |
| 88 | Having people who support us is important! Think about who in your life can help you stick with the goals that are important to you. | Social support (unspecified; 3.1) |
| 89 | Your first plan will not work 100% of the time. Consider changing your goals until you find what works best for you! | Review outcome goals (1.7) |
| 90 | Think about what small steps you can take to reach your movement goals. | Goal setting (behaviour; 1.1) |
| 91 | Some people find it easier to make small changes to their daily routine. Think about a plan to make a small change that would work for you this week. | Action planning (1.4) |
| 92 | There will be times when you don't achieve your goals. This happens to everyone! Think about how you can respond to these times in a productive way. | Problem solving (1.2) |
| 93 | When you make a plan, think about when, where, and how often you want to do a certain behavior. | Action planning (1.4) |
| 94 | Think about a plan for what you want to do to reach your movement goals this week. The more detailed the plan, the more likely you are to do it! | Action planning (1.4) |
| 95 | Tracking your physical activity can help you learn about your own behaviors. Check in on your progress in the REACH app! | Self-monitoring of behaviour (2.3) |
| 96 | Tracking your physical activity can take time, but it can make you feel more accountable and motivated! Check in on your progress in the REACH app! | Problem solving (1.2) |
| 97 | Maintaining active behaviors can be challenging. Many people find it helpful to share this journey with a close friend. | Social support (unspecified; 3.1) |
| 98 | It can take a lot of trial and error to find what movement works. Many people feel that knowing what doesn't work for them is as valuable as knowing what does! | Behavioural experiments (4.4); Social comparison (6.2) |
| 99 | People who make changes that they enjoy are more likely to maintain them in the long run. | Information about social and environmental consequences (5.3); Social comparison (6.2) |
| 100 | Becoming more physically active may not always be easy. What are some reasons for you to stick with it? | Information about social and environmental consequences (5.3); Info about emotional consequences (5.6); Pros and Cons (9.2) |
| 101 | Reminders can be helpful when we are trying to form new habits. You can put a reminder in your phone, a note on your fridge, or leave yourself a voice message! | Prompts/cues (7.1) |
| 102 | Making small changes that build over time is more effective than one big change you can’t stick with. What are some small movements you can add to your day? | Graded tasks (8.7) |
| 103 | Research shows tracking your daily movement is an important part of changing behavior for many people. You can log your activity in the REACH app! | Self-monitoring of behaviour (2.3); Credible source (9.1) |
| 104 | Think about all the great reasons why you are making these changes. These reasons likely outweigh the cons! | Pros and cons (9.2) |
| 105 | Change is challenging. Remember to take time to reflect on your dedication - be proud of yourself! | Focus on past success (15.3) |
| 106 | Don't forget to celebrate the progress you've made in achieving your movement goals! | Non-specific reward (10.3); Self-reward (10.9); Reward approximation (14.4) |
| 107 | Reflect on the progress you've made. You will surprise yourself on how far you've come! | Focus on past success (15.3) |
| 108 | Try taking a moment to think about all the hard work you've put in. You are becoming a more active you! | Social support (unspecified; 3.1); Focus on past success (15.3) |
| 109 | Planning ahead can help reduce stress when you're busy. Consider scheduling some workouts for this week! | Reduce negative emotions (11.2); Conserving mental resources (11.3) |
| 110 | Set yourself up for success. Small changes to your home or work can help you succeed in achieving your goals. | Restructuring the physical environment (12.1); |
| 111 | You've worked hard and proved to yourself what you are capable of. Take pride in your daily achievements. You can inspire others, too! | Social support (unspecified; 3.1); Identification of self as role model (13.1) |
| 112 | The steps you take towards staying active can motivate those around you! | Identification of self as role model (13.1) |
| 113 | Not every part of changing behaviors is fun. Try to focus on the positive aspects of making these changes (e.g., improved mood, more energy)! | Framing/reframing (13.2) |
| 114 | Don’t think of a slip as harmful or bad; they are INEVITABLE. Don't be discouraged! What is more important is how we react to slips and recommit to change. | Framing/reframing (13.2) |
| 115 | Celebrate all the progress you've made, the goals you've reached, and the goals you will conquer! | Non-specific reward (10.3); Self-reward (10.9); Reward approximation (14.4) |
| 116 | Try taking some time to think about and reflect on how you feel about each new change you've made. | Focus on past success (15.3) |
| 117 | Try reflecting on and celebrating each new change you've made. | Non-specific reward (10.3); Self-reward (10.9); Reward completion (14.5) |
| 118 | Changes to a more active lifestyle are exciting and hard. Continue to succeed in these pride-worthy changes! | Verbal persuasion about capability (15.1) |
| 119 | When you put your mind to something, you can do it! | Verbal persuasion about capability (15.1) |
| 120 | You can make lasting changes to improve your energy levels! | Verbal persuasion about capability (15.1) |
| 121 | Think about all the great changes that you have made since joining the program. Every small step you take along the way counts! | Focus on past success (15.3) |
| 122 | You are making changes that will benefit your overall health and lifestyle. Remind yourself of these benefits. | Self-talk (15.4) |
| 123 | Remind yourself why having an active lifestyle is important. Keep these reasons in mind as you go about your day! | Self-talk (15.4) |
| 124 | Think about what movement goals you want to make for this week, and don’t forget to log them in the REACH app! | Goal setting (outcome; 1.3) |
| 125 | Think about a goal you could make to be more physically active this week. | Goal setting (outcome; 1.3) |
| 126 | What is a goal you can make to be active this week? You can use the REACH app to log your goal. | Goal setting (outcome; 1.3) |
| 127 | Think about some ways you can fit physical activity into your schedule when you have a busy week. | Problem solving (1.2) |
| 128 | Each season has different challenges to staying active. Think about ways you can exercise when the weather changes. | Problem solving (1.2); Action planning (1.4) |
| 129 | Think about where, when, and how you'll get your physical activity in today! | Action planning (1.4) |
| 130 | Try aiming for medium intensity movement. Use the talk test to monitor intensity. High= it’s hard to talk. Medium = it’s hard to sing. | Self-monitoring of behaviour (2.3) |
| 131 | We all need support from others to help us with our goals sometimes. Think about who in your life can help you stick to your workout plan. | Social support (unspecified; 3.1) |
| 132 | Working out with a buddy can give you motivation and support. It can also be a nice way to spend time together! | Social support (practical; 3.2) |
| 133 | Scheduling your movement sessions like an appointment can help you stay on track and get your workouts in! | Action planning (1.4); Prompts/cues (7.1) |
| 134 | What are some ways you can stay active? Movement has many benefits, like more energy and better sleep. | Information about health consequences (5.1) |
| 135 | Pay attention to how you're feeling during physical activity, so you know how hard you're working. Don't forget to pace yourself. | Monitoring of emotional consequences (5.4) |
| 136 | Some people find it challenging to stick to a new physical activity routine. Think about some good reasons to stick to yours! | Social comparison (6.2) |
| 137 | You can reduce your sitting time by going for a walk after dinner. Just a 15-minute walk can make a difference! | Behavioural substitution (8.2); Habit reversal (8.4) |
| 138 | You can make physical activity a part of your routine by linking it to something you do daily. For example, consider going for a walk or bike ride after dinner. | Behavioural practice/rehearsal (8.1); Habit formation (8.3) |
| 139 | Reversing old habits can be hard, so start small. Try taking the stairs instead of the elevator, park at the far end of a parking lot, or commute by walking! | Behavioural substitution (8.2); Habit reversal (8.4) |
| 140 | If you have missed a few workout sessions, it's okay! Try starting with a shorter session and work your way up over time. | Graded tasks (8.7); potentially Social support unspecified (3.1) |
| 141 | National health guidelines encourage everyone, including people with lupus, to get 150 minutes or more of physical activity per week. You can get there! | Social support (unspecified; 3.1); Instruction on how to perform the behaviour (4.1); Credible source (9.1) |
| 142 | For many people, regular movement can decrease stress and improve their mood. | Information about emotional consequences (5.6) |
| 143 | Need a distraction to get through your workout session? Some people like to listen to music/podcasts or workout with a buddy! What works for you? | Restructuring the physical environment (12.1); Restructuring the social environment (12.2); Distraction (12.4) |
| 144 | Some people find that scheduling in their daily movement can help reduce stress. | Reduce negative emotions (11.2); Conserving mental resources (11.3) |
| 145 | Putting your workout clothes and shoes near the front door can serve as a reminder to get your exercise in! | Prompts/cues (7.1) |
| 146 | There are some weeks when reaching your movement goal may seem daunting. In those weeks, try thinking about reducing the time you spend sitting each day. | Framing/reframing (13.2) |
| 147 | Staying active is not an all-or-nothing approach. If you partially complete your goal this week it will bring you closer to your long-term movement goals. | Social support (unspecified) (3.1); Verbal persuasion about capabilities (15.1) |
| 148 | Every workout session you complete gets you closer to your long-term goals. Remember to celebrate the small steps you're making along the way! | Non-specific reward (10.3); Reward approximation (14.4) |
| 149 | You have the ability to live an active lifestyle. You've already shown yourself how! Remember how capable you are if you start to doubt your abilities. | Verbal persuasion about capability (15.1); Focus on past success (15.3) |
| 150 | You've shown yourself that you can integrate movement into your daily routine. Keep up the great work! | Social support (unspecified; 3.1); Focus on past success (15.3) |
| 151 | You've shown yourself that you are capable of being active! Look at your progress in the REACH app to see how far you’ve come! | Focus on past success (15.3) |
| 152 | We all feel different physical sensations while working out. When you start to sweat or breath heavy, remind yourself that it is a normal part of being active. | Self-talk (15.4) |
| 153 | Think about all the positive benefits you will get during your next workout. Better mood, sleep, reduced fatigue! | Imaginary reward (16.2); Information about health consequences (5.1) |
| 154 | Consider swapping a behavior you want to change with one that aligns with your goals. For example, I take the stairs instead of the elevator to fight fatigue. | Behaviour substitution (8.2) |

MacPherson, M., Cranston, K., Johnston, C., Locke, S., & Jung, M. E. (2021). Evaluation and Refinement of a Bank of SMS Text Messages to Promote Behavior Change Adherence Following a Diabetes Prevention Program: Survey Study. *JMIR Formative Research*, *5*(8), e28163. https://doi.org/10.2196/28163

MacPherson, M. M., Cranston, K. D., Locke, S. R., Bourne, J. E., & Jung, M. E. (2021). Using the behavior change wheel to develop text messages to promote diet and physical activity adherence following a diabetes prevention program. *Translational Behavioral Medicine*, *11*(8), 1585–1595. https://doi.org/10.1093/tbm/ibab058

Michie, S., Richardson, M., Johnston, M., Abraham, C., Francis, J., Hardeman, W., Eccles, M. P., Cane, J., & Wood, C. E. (2013). The Behavior Change Technique Taxonomy (v1) of 93 Hierarchically Clustered Techniques: Building an International Consensus for the Reporting of Behavior Change Interventions. *Annals of Behavioral Medicine*, *46*(1), 81–95. https://doi.org/10.1007/s12160-013-9486-6
